# Quality and safety requirements for pharmacy-based vaccination in resource-limited countries

**DOI:** 10.1186/s41182-025-00698-5

**Published:** 2025-02-06

**Authors:** Kidanemariam G/Michael Beyene, Melaku Tileku Tamiru

**Affiliations:** 1 Ethiopian Food and Drug Authority, Addis Ababa, Ethiopia; 2 College of Health Sciences, Department of Pharmacology and Clinical Pharmacy, Addis Ababa University, Addis Ababa, Ethiopia

**Keywords:** Pharmacist role, Public health, Pharmacy-based vaccination, Resource-limited countries, Vaccination coverage

## Abstract

Pharmacy-based vaccination (PBV) programs have proven to be a promising strategy for improving vaccination coverage, particularly in resource-limited countries. These programs increase accessibility and convenience, increase vaccination rates, and benefit vulnerable populations. However, successful implementation requires addressing gaps in regulatory oversight, pharmacists training, inter-professional collaboration, and public awareness. With proper regulatory frameworks, advocacy, enhanced training programs, public education, and establishment of well-designed database, PBV can achieve outcomes comparable to high-resource settings. This commentary aims to inform stakeholders and offer practical recommendations to minimize risks while leveraging its benefits.

## Introduction

Vaccination is one of the most effective public health strategies for preventing infectious diseases, yet many resource-limited countries often face significant barriers to reaching optimal vaccination coverage [1]. In countries like Ethiopia, vaccination coverage is low, dropout rates are high, and access among marginalized population is limited [2, 3]. The global push toward achieving the Sustainable Development Goals (SDGs)—a set of 17 goals adopted by the United Nations in 2015 to address global challenges, such as poverty, health disparities, and inequality by 2030—has brought innovative solutions to forefront [4]. These goals emphasize universal health coverage and equitable access to essential services, including vaccinations.

Pharmacy-based vaccination (PBV) has emerged as one such solution, offering increased accessibility, convenience, and improved vaccination rates. However, successful implementation requires addressing barriers specific to resource-limited settings. This commentary emphasizes the potential benefits of PBV program in resource-limited settings while addressing challenges that can be mitigated through proper regulatory oversight, training, and inter-professional collaboration. With robust measures in place, PBV can significantly improve vaccination rates, particularly among underserved populations, as demonstrated in countries where these programs are well established.

### Accessibility and convenience

Pharmacy-based vaccination (PBV) enhances accessibility by leveraging pharmacies’ extended hours and walk-in availability, making them a convenient option for underserved communities. PBV programs have significantly improved vaccination rates, especially among vulnerable populations, as seen in high-resource settings. For example, studies in USA and Canada have shown that PBV programs effectively increase immunization rates while maintaining high safety standards [4, 5], However, in resource-limited settings, regulatory frameworks ensure quality and safety, particularly in vaccine storage and administration [5, 6]. For example, maintaining the cold chain for vaccines requires a reliable power supply, which may be inconsistent in many regions. Hygienic practices during vaccine administration are equally critical to ensure patient safety. It is therefore essential to pilot and implement the stated requirements in selected pharmacies, followed by phased scaling with uniform regulations and standards applied across the country.

### Service remuneration

Remuneration for pharmacy vaccination services is a key policy enabler for PBV [5]. However, inconsistent reimbursement models across countries regarding service fees, eligible vaccines, and targeted populations significantly affect the uptake and effectiveness of these programs [7–9]. Reviews highlighted the importance of well-structured payment systems to sustain PBV programs [8, 9]. Properly designed policies and legislation must be developed before commencing PBV, especially in resource-limited settings to ensure that offering PBV is financially viable for both pharmacies, users and funders.

### Training and competency standards

The effectiveness of PBV relies heavily on pharmacists being well trained in vaccine administration and the management of adverse reactions. Many resource-limited settings lack accredited training programs for this purpose. Standardized, competency-based accredited training programs, as implemented in countries like USA and Canada, can serve as a model [6, 10, 11]. Tailoring these programs to the resource-limited context, while ensuring alignment with global standards, is crucial for successful implementation [10, 12].

### Opposition from other healthcare professionals

Opposition from some healthcare professionals often reflects outdated or paternalistic perspectives rather than evidence-based concerns. For example, medical professionals in German, Italy, Greece, and Jordan have opposed authorizing pharmacists to administer vaccines, citing concerns about their competence in managing clinical histories, diagnosing conditions, and addressing adverse effects [13, 14]. However, studies consistently demonstrate that PBV programs are safe and effective, particularly when supported by training and regulatory oversight [15–18]. For example, an on-site pharmacist-led vaccination service in an assisted-living facility demonstrated high safety standards [19]. Similarly, pharmacist-managed vaccination programs have significantly increased influenza vaccination rates among cardiovascular patients without compromising safety [18]. Encouraging inter-professional dialog and establishing clear legislative support for pharmacists’ role in vaccination, backed with accredited training, can mitigate such opposition [13, 20, 21].

### Public education, perception, and vaccine hesitancy

Pharmacists play an important role in educating the public about vaccine safety and efficacy [11, 22]. However, in resource-limited countries, skepticism about pharmacists’ qualifications may hinder vaccination uptake. Cultural beliefs, past negative experiences, and misinformation further contribute to vaccine hesitancy [23, 24]. Effective public education campaigns, leveraging social media and community outreach can counter misconceptions. In addition, highlighting pharmacists’ adherence to rigorous training and safety standards can build public trust in PBV services [25].

### Impact on vulnerable populations

PBV programs can benefit vulnerable populations, such as the elderly and low-income individuals, who face barriers to accessing healthcare services [26, 27]. Strong regulatory oversight and quality control mechanisms ensure that these populations receive safe and effective care. Ensuring public awareness of pharmacies’ compliance with rigorous quality standards will support the gaining of trust of vulnerable groups and improve vaccination rates. Pharmacists should also be trained on how to effectively communicating with patients about vaccines, including strategies for addressing vaccine hesitancy.

### Regulatory challenges

Weak or poorly enforced regulations can lead to serious public health risks, including the distribution of substandard and falsified vaccines. Strong regulatory oversight, coupled with regular audits and feedback mechanisms, is essential to ensure that pharmacies adhere to national vaccination standards. This improves the credibility and safety of PBV programs [6, 28].

### Integration with public health strategies

To maximize the potential of PBV, it must be integrated into broader public health strategies. A centralized vaccination database accessible by pharmacies and other healthcare providers is essential for documenting administered vaccines, ensuring continuity of care, preventing duplicate vaccinations, and enhance tracking and sharing of vaccination data [29, 30]. In Alabama, for example, voluntary provider participation in the state vaccine information system leads to incomplete vaccination records [31]. Such system can enhance coordination, track immunization coverage, and vaccine management, and improve patient outcomes, particularly in resource-limited settings where record-keeping may be fragmented [30–32].

Collaboration among pharmacies, health professionals, public health organizations, and healthcare facilities is essential to ensure that PBV aligns with national health goals and contributes to the broader public health agenda [14, 22, 32]. It can also enhance the reach of these programs and ensure consistent service delivery. It is also important to establish a firm and evidence-based position among relevant stakeholders regarding the role of pharmacists as vaccinators, to avoid controversy and debate over this scope of practice [13].

### Challenges in maintaining standards

Even in settings where laws and guidelines are in place, maintaining high standards can be challenging. Issues, such as staff turnover, variations in the enforcement of regulations, and limited resources, can hinder the effectiveness of PBV programs [6, 33]. Continuous training, regular audits, and strong regulatory frameworks can ensure sustained quality and safety of the vaccination services [6, 14].

### Outlook

PBV offers a promising solution for increasing vaccination coverage in resource-limited settings; however, planning and investment are required to ensure that pharmacists adhere to the highest standards of care that are supported by strong legal and regulatory frameworks, are appropriately remunerated for the services and vaccines, and receive ongoing training. Establishing clear legislation, attaining stakeholder consensus, and providing public education on the role of pharmacists in vaccination is essential to the success of these programs. Future research should focus on optimizing program implementation, evaluating the long-term impacts, and developing scalable models tailored to resource-limited contexts.

## Data Availability

No datasets were generated or analyzed during the current study.
